# The Hemodynamic Effects of Interatrial Septostomy in Iatrogenic Mitral Stenosis Following MitraClip

**DOI:** 10.1016/j.jscai.2024.102019

**Published:** 2024-05-18

**Authors:** Evan Harmon, Benjamin Zorach, Serge Harb, Samir Kapadia, Grant Reed

**Affiliations:** Department of Cardiovascular Medicine, Cleveland Clinic Foundation, Cleveland, Ohio

**Keywords:** interatrial septostomy, mitral valve disease, mitral valve repair/replacement

## Abstract

Iatrogenic mitral stenosis is a rare complication of the MitraClip procedure for which limited therapeutic options exist. We present a unique case using real-time echocardiographic and hemodynamic data demonstrating a potential role for interatrial septostomy creation as a percutaneous management option in this challenging population.

## Introduction

Transcatheter mitral valve edge-to-edge repair with the MitraClip system (Abbott) is a safe and effective means of treating severely symptomatic patients with primary mitral regurgitation (MR) at high surgical risk, as well as select patients with secondary MR who remain symptomatic despite optimal medical therapy for heart failure.^1^ An important potential complication of the MitraClip procedure is iatrogenic mitral stenosis (MS), which may result in poor postprocedural outcomes.^2^^,^^3^ Currently, the only definitive management strategy for iatrogenic MS is high-risk mitral valve surgery. In select patients, however, interatrial septostomy (IAS) creation via transseptal puncture may attenuate the deleterious hemodynamic effects of iatrogenic MS and provide symptom palliation.

## Case report

A 70-year-old man with a history of severe MR, nonischemic cardiomyopathy (ejection fraction 35%-40%) status post primary prevention dual-chamber implantable cardioverter-defibrillator (ICD), paroxysmal atrial fibrillation, chronic kidney disease, and multiple myeloma on chemotherapy was referred to our institution for possible redo transcatheter mitral valve repair. Two years prior to presentation, he had undergone transcatheter edge-to-edge repair (TEER) at an outside facility with the MitraClip system, with an XTR clip deployed in the A2/P2 position, and an NTR clip deployed in the A1/P1 position.

**Figure 1:**
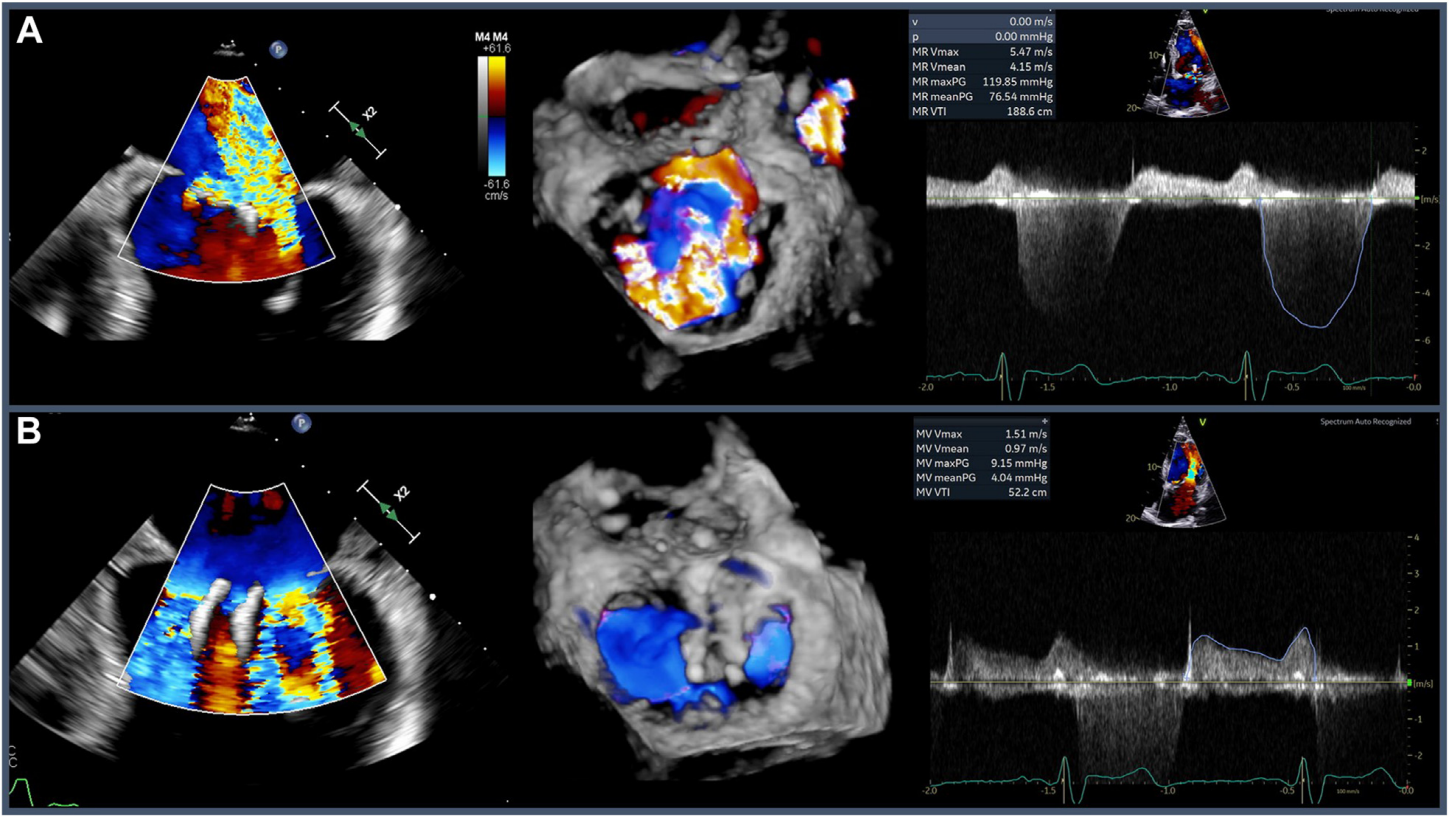
**Pre-procedure transesophageal echocardiogram.** (**A**) Transesophageal echocardiogram bicommissural view at 60 degrees of the mitral valve with color Doppler demonstrating severe mitral regurgitation (MR) with both lateral and medial jets. Accompanied by 3D reconstruction and transthoracic echocardiogram pulse wave Doppler demonstrating holosystolic regurgitation with a maximum velocity of 5.47 m/s. (**B**) Transesophageal echocardiogram bicommissural view at 60 degrees of the mitral valve with color Doppler demonstrating turbulent flow across the valve in the setting of mitral stenosis. Accompanied by 3D reconstruction and pulse wave Doppler measuring peak gradient of 9 mm Hg and a mean gradient of 4 mm Hg.

In the interim, he had developed New York Heart Association (NYHA) class III symptoms despite maximally-tolerated medical therapy, and transesophageal echocardiography demonstrated severe (4+) holosystolic MR with a predominant laterally originating jet and smaller medial jet despite stable placement of his prior clips. He was also noted to have moderate MS with peak and mean gradients of 9 and 4 mm Hg, respectively (Figure 1). Given his mixed mitral valve disease, a multidisciplinary heart team discussion was held and the patient was deemed at high surgical risk for mitral valve replacement (MVR). The decision was made to attempt redo MitraClip XTR system placement between the 2 prior clips in an effort to reduce MR severity while minimally impacting his known moderate MS.

Intraprocedurally, the initial left atrial pressure was noted to be 12 mm Hg with V-waves to 16 mm Hg following transseptal puncture. A 4F multipurpose catheter was positioned in the left upper pulmonary vein for continuous hemodynamic monitoring. After serial dilations of the interatrial septum, a 24F steerable sheath was advanced into the left atrium through which the MitraClip XTR system was delivered. However, this XTR clip could not be deployed between the prior clips as originally planned due to their orientation and endothelialization. Attempted clip placement at the lateral orifice did not significantly reduce the patient’s MR and, as anticipated, slightly worsened his MS (peak gradient 14 mm Hg, mean gradient 7 mm Hg). Thus, clip deployment was felt to be infeasible and was aborted.

**Figure 2:**
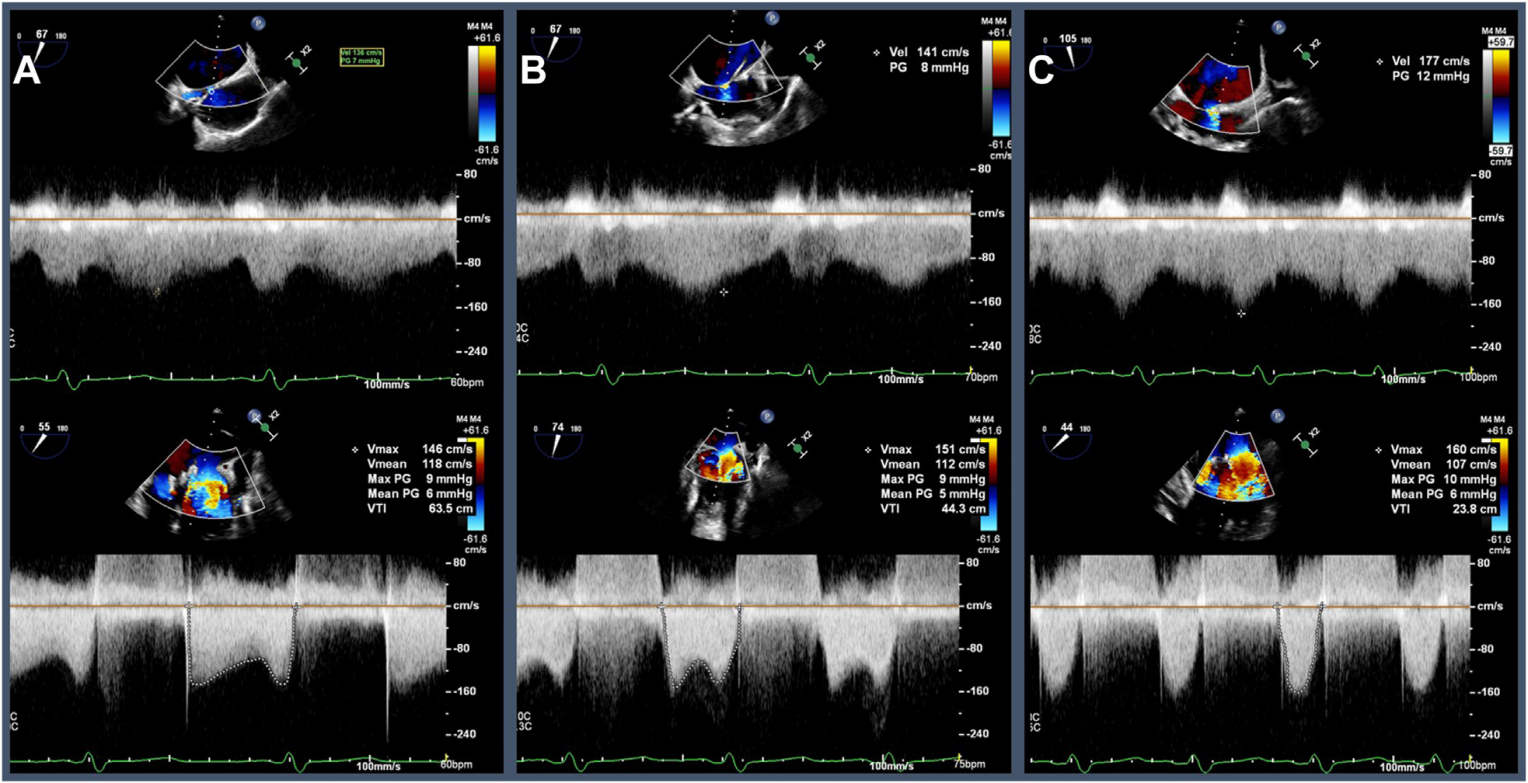
**Intraprocedural transesophageal echocardiogram pulse wave Doppler tracings across IAS (top panels) and mitral valve (bottom panels).** (**A**) HR 60 bpm, mean MV gradient 6 mm Hg, IAS velocity 136 cm/s. (**B**) HR 75 bpm, mean MV gradient 5 mm Hg, IAS velocity 141 cm/s. (**C**) HR 100 bpm, mean MV gradient 6 mm Hg, IAS velocity 177 cm/s.

**Figure 3:**
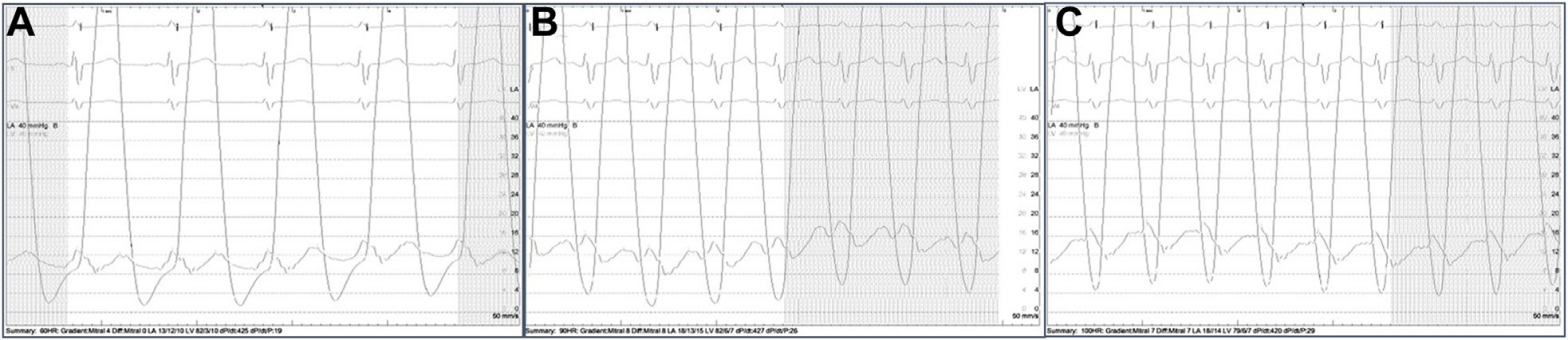
**Left atrial (LA) and ventricular waveform tracings at increasing heart rates.** (**A**) Mean LA pressure 10 mm Hg at HR 60 bpm. (**B**) Mean LA pressure 15 mm Hg at 90 bpm. (**C**) Mean LA pressure 14 mm Hg at 100 bpm.

We did wonder, however, whether the IAS created during transseptal puncture had modified the hemodynamic consequence of the patient’s MS. At a baseline paced rhythm with heart rate (HR) of 60 bpm, the mean transmitral gradient was measured to be 6 mm Hg with velocity across the IAS 136 cm/s. The patient’s pacing rate was increased in a stepwise fashion to 75 bpm, with a mean gradient across the mitral valve measuring 5 mm Hg and velocity across the IAS 141 cm/s. The HR was again increased to 100 bpm, with a mean gradient of 6 mm Hg, and IAS velocity of 177 cm/s (Figure 2). The left atrial pressure was noted to remain relatively unchanged despite increasing HR with mean pressures of 10 mm Hg, 15 mm Hg, and 14 mm Hg corresponding to rates of 60, 90, and 100 bpm, respectively (Figure 3). The case was then concluded with the hope that the patient’s IAS may serve as a bridge to high-risk MVR.

A limited transthoracic echocardiogram performed postprocedure day 1 demonstrated moderately severe MR (3+), peak and mean MV gradients of 9 and 6 mm Hg (HR 63 bpm), and normal right ventricular size and function. Despite suffering a setback in the form of left foot osteomyelitis in the context of his chemotherapy-related immunosuppression, his heart failure symptoms remained stable over the following 2 months and a follow-up echocardiogram at that time confirmed the persistent patency of his IAS with left-to-right shunting detected by color Doppler. His mitral valve gradients (peak 10 mm Hg, mean 5 mm Hg, HR 62 bpm) as well as his right ventricular size and function, remained stable. He was then able to undergo successful high-risk MVR and IAS closure with improvement in symptoms and functional capacity.

## Discussion

Limited therapeutic options exist for symptomatic patients with moderate-to-severe MS following TEER at high surgical risk. A primary driver in the pathophysiology of MS is left atrial pressure overload as a hemodynamic consequence of the increasing diastolic pressure gradient across a stenotic mitral valve. One mechanism by which elevated left atrial pressure may be partially relieved is via IAS creation with subsequent left-to-right shunting serving as a “pop-off valve.”

The coexistence of atrial septal defect and MS has been referred to as Lutembacher syndrome, with the observed atrial septal defect appearing to have an initial protective role in MS by avoiding hydrostatic overload of the pulmonary venous system and subsequent pulmonary congestion.^4^ This raises the question of whether IAS may in fact be a mechanism by which symptomatic relief could be provided to patients with severe MS.

Our case utilizes real-time echocardiographic and hemodynamic data to uniquely demonstrate this hypothesized protective effect of IAS creation in a patient with mixed mitral valve disease following TEER. The maintenance of a stable mitral valve gradient and left atrial pressure despite increasing HR was mediated by left atrial unloading and increasing flow across the IAS, serving as a bridge to high-risk surgical MVR.

## Conclusion

Given the limited treatment options for patients with symptomatic MS at high surgical risk, there may be a role for dedicated clinical trials exploring percutaneous IAS creation as a potential therapeutic intervention in high-risk surgical patients with moderate-to-severe MS.
